# Mapping Current Studies of tRNA Fragments onto Disease Landscape

**DOI:** 10.3390/biom15040512

**Published:** 2025-04-01

**Authors:** Sathyanarayanan Vaidhyanathan, MacKenna Durbin, Adesupo A. Adetowubo, Lisa H. Do, Sheida Kavehmoghaddam, Sai Anusha Jonnalagadda, Bryan Ramirez Aguilar, Tamin Ortiz-Gomez, Yan X. Lin, Asim Dave, Fatmanur Kiliç, Alexa R. Karp, Mohammed Imthiyas Rahmah, Noor F. Riaz, Nikhila Mandava, Aleece Siner, Andrey Grigoriev

**Affiliations:** 1 Department of Biology, Rutgers University, Camden, NJ 08102, USA; sv646@scarletmail.rutgers.edu (S.V.); md1842@scarletmail.rutgers.edu (M.D.); aaa586@scarletmail.rutgers.edu (A.A.A.); lhd26@scarletmail.rutgers.edu (L.H.D.); sk2673@scarletmail.rutgers.edu (S.K.); sj1110@scarletmail.rutgers.edu (S.A.J.); bgr25@scarletmail.rutgers.edu (B.R.A.); tno7@scarletmail.rutgers.edu (T.O.-G.); fk274@scarletmail.rutgers.edu (F.K.); ark230@scarletmail.rutgers.edu (A.R.K.); nfr22@scarletmail.rutgers.edu (N.F.R.); nm1216@scarletmail.rutgers.edu (N.M.); ass224@scarletmail.rutgers.edu (A.S.); 2 Center for Computational and Integrative Biology, Rutgers University, Camden, NJ 08102, USA; yxl1@scarletmail.rutgers.edu (Y.X.L.); ad2119@scarletmail.rutgers.edu (A.D.); rm1940@scarletmail.rutgers.edu (M.I.R.)

**Keywords:** transfer RNA, transfer RNA fragments, regulation, biomarkers, cancer, neurological diseases, cardiovascular diseases, musculoskeletal diseases

## Abstract

Transfer-RNA-derived fragments (tRFs) are a relatively recently discovered class of non-coding RNAs derived from both precursor and mature transfer RNAs (tRNAs). Research on these molecules has been expanding rapidly, revealing their diverse roles in cellular processes, both in normal physiology and in disease states, often via post-transcriptional regulation of target genes. Altered tRFs abundances have been implicated in various conditions, where they may act as either drivers of disease progression or as protective agents. For instance, specific tRFs are associated with increased risk for cancer metastasis, while others may suppress tumor cell proliferation. Despite the growing recognition of tRFs as functional RNAs rather than sequencing noise, this field of study faces numerous challenges. Inconsistent naming conventions and variability in experimental approaches hinder the comparison of findings across studies, limiting our understanding of the common roles and mechanisms of tRFs. This review provides a comprehensive analysis of current literature on the various roles of tRFs in different diseases, particularly focusing on four broad areas: cancer, neurological, cardiovascular, and musculoskeletal disorders. We analyze studies that link specific tRFs to various aspects of human diseases and provide a convenient classification of these studies regarding the depth of the provided evidence. Further, we note gaps in current investigations and consider strategies to address methodological inconsistencies, including validation experiments and unified nomenclature. By consolidating research in this manner, we aim to facilitate comparisons across diverse studies, enhancing our ability to identify functional commonalities and furthering our understanding of the mechanisms by which tRFs act.

## 1. Introduction

Transfer-RNA-derived fragments (tRFs) represent a novel class of small RNA molecules that have recently been identified due to advances in sequencing technologies. This discovery has expanded our understanding of tRNAs, traditionally known for their roles in translating mRNA codons into amino acids. The human genome harbors over 600 tRNA genes, far exceeding the number of possible codons, hinting at additional roles for tRNA in various cellular functions and processes.

**Figure 1:**
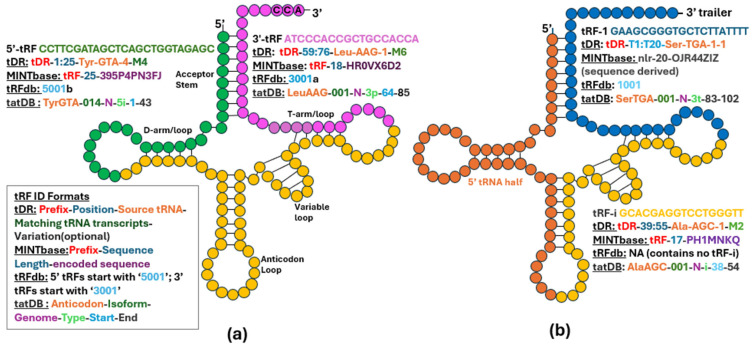
Naming conventions of tRFs. Mature tRNA with a CCA end is shown schematically in (**a**) and precursor tRNA with a 3′-end trailer—in (**b**). Sequence colors match those of the tRNA segments, from which the respective examples of tRFs originate, together with their names produced by different naming schemes (see text): (**a**) tRF-5p (green) and tRF-3p (purple). The box in the bottom–left spells out the naming formats used for tRFs by various databases; the color coding within the IDs below each tRF reflects these formats. The tRF ID for the 3′tRF example is shown in purple, while that for a 5′ tRF example is displayed in green. Names of the different regions of a tRNA are given in smaller bold font near their corresponding locations in the structure. (**b**) tRNA half (orange), tRF-1, or 3′trailer tRF (blue), and tRF-i (yellow). A sequence/naming example is not provided for the tRNA half, as their names are similar to shorter 5′ and 3′tRFs.

A tRNA gene is transcribed by RNA polymerase III into a precursor, which undergoes several modifications and is further cleaved to form a mature tRNA. Mature tRNAs are typically fewer than 95 nucleotides (nts) in length, and their secondary structure resembles a clover leaf. A properly folded tRNA normally has an acceptor stem, a D-arm/loop, a T-arm/loop, an anticodon loop, and a variable loop region. Despite having different sequences, all tRNAs have the same characteristic cloverleaf structure (Figure 1). Mitochondrial tRNAs may deviate in some of the above aspects, typical for nuclear-encoded tRNAs.

tRFs are produced from both precursor and mature tRNAs through direct cleavage by ribonuclease (RNase) enzymes, including Angiogenin (ANG) and RNase Z, often under stress conditions [1,2]. DICER, a protein known for cleaving precursor microRNAs (miRNAs), also plays a role in the biogenesis of tRFs by processing them near the D-loop [3]. However, DICER was shown to be essential for producing only a subset of tRNAs [3,4,5,6,7,8], and ANG-induced cleavage is also selective [9]. tRFs can also be produced in the absence of stress conditions, e.g., through mechanisms such as hormone signaling [10]. These findings suggest that there are other RNases and mechanisms responsible for the biogenesis of tRFs which have yet to be discovered. However, in this review, we are not focusing on the biogenesis of these molecules (well described elsewhere [11]).

The initial tRF report [2] described several distinct types of tRFs based on their location within the tRNA: tRF-5, tRF-3 (Figure 1a), and tRF-1 (Figure 1b). tRF-5 and tRF-3 are derived from the extreme 5′ and 3′ ends of mature tRNAs, respectively, and their sequences extend roughly to the nearest loop. If they extend to the anticodon loop, then they are usually called tRNA halves (Figure 1b). Most other “intermediate” fragments are called tRF-i (Figure 1b). However, naming conventions for tRFs vary significantly across research papers, often not aligning with those used in the initial tRF report. Also, various terms, such as tsRNAs, tiRNAs, tDRs, tsncRNA, and others are used interchangeably to refer to tRFs, without providing any additional meaning. To avoid unnecessary confusion, we simply refer to all these fragments as tRFs, regardless of the terms used in the original papers.

Additionally, there is a wild inconsistency in the naming of specific tRFs. The sequences of tRFs can be assumed to be unique identifiers. However, strictly speaking, it is still unclear which tRNA modifications retained or produced during their biogenesis affect tRF function and how this should be represented in the name. A relatively short tRF sequence may be a good candidate for a name (so that a simple copy–paste right from a paper’s text could allow quick sequence searches) but would look somewhat unwieldy in manuscripts. Therefore, several naming approaches have been undertaken.

For instance, the tRF database (tRFdb) [12] assigns tRF IDs, such as 1001, 1002… to tRF-1; 3001, 3002… to tRF-3; and 5001, 5002… to tRF-5. This method generates the shortest names, but it limits the repertoire of tRFs only to those included in tRFdb and ignores others, often whole classes, such as tRF-i (Figure 1b). Some research papers base their analysis on exact matching of the tRF molecules they sequenced to tRFdb entries, which could lead to missing relevant tRFs in their results.

In contrast, a recently reported tool called tDRnamer generates standardized five-part names for tRFs based on their sequences [13]. These names consist of three mandatory and two optional components for special cases. Each generated tRF name begins with the prefix “tDR”, followed by the position in the source tRNA indicated as (start:end) and the source tRNA itself as a required component (Figure 1). Yet, identifying the correct source tRNA is challenging because different tRNAs may harbor identical tRFs. Additionally, multiple isodecoders (tRNAs with the same anticodon but from different genomic loci) exist for a single isoacceptor (tRNAs that can accept the same amino acid regardless of the anticodon). With tDRnamer, the optional naming components provide additional information, such as the number of source tRNA genes that could generate a specific tRF sequence and any variations present in the sequence. However, this has some shortcomings; in particular, the names may point to a plausible but potentially incorrect origin of the sequence (being from another source tRNA gene), and the names may also be just as long as the tRF sequences themselves, making them equally unwieldy. It is important to note that direct comparisons of tDRnamer with tRFdb are not correct, as tRFdb is a database of a subset of experimentally observed tRFs, and tDRnamer is a tool, mapping an arbitrary sequence input to a possible name.

Another database, MINTbase [14], belongs to both categories. It contains a list of all tRFs detected in a large set of small RNA-sequencing datasets as well as an automated short RNA nomenclature system (named “license plates”) that attempts to cover all possible short sequences, including truncated subsequences (matching parts of tRNAs) present in the genome. This nomenclature uses a three-part format, consisting of a prefix, the tRF sequence length, and the encoded sequence, along with alternative MINTbase IDs based on genomic location (Figure 1). This naming system also produces rather short names, but as is the case with the tRFdb, they may sound somewhat enigmatic for readers.

One of the mechanisms of action of tRFs found in multiple organisms is in silencing genes by interacting with Argonaute (AGO) proteins, forming the RNA-induced silencing complex (RISC). It was hypothesized that tRFs were an important step in the origin of RISC [15]. This complex enables tRFs to initiate seed-base pairing and bind to targets, resembling the interaction between miRNAs and their targets. Previous findings from our lab reported on tRFs and their mRNA targets through computational analysis, revealing potential binding patterns for hundreds of AGO-loaded tRFs [16,17], obtained from large-scale Cross-Ligation and Sequencing of Hybrids (CLASH) experiments [18].

Having compiled such data together, we previously described a database of tRF targets (tatDB) that includes ~250,000 experimentally determined tRF-target pairs [19]. This database contains the secondary structures of these hybrids and binding motifs present in tRFs to illustrate their potential modes of interaction, essential for understanding the targeting mechanisms of tRFs. tatDB names tRFs using the format AA_anticodon-Isoform-Genome-Type-Start-End (Figure 1). As with the tRDnamer, such naming is not unique, as tRFs with identical sequences may be generated from different tRNAs. However, tatDB and tRDnamer provide the most informative names regarding the origin of most tRFs.

### Examples of Mechanisms and Processes Involving tRFs

Other mechanisms of tRF action have also been reported (in addition to RISC-based ones), and we list some of them briefly here. Certain tRFs have been reported to assemble into G-quadruplex structures or to exhibit translation inhibition activity. RNA modifications obtained in vivo have been reported to confer greater inhibitory activity to endogenous tRFs compared to their synthetic counterparts [20]. Thus, changes to tRNA modifications may affect their function and, in turn, influence disease mechanisms. The same is likely true for changes in tRF biogenesis, since that would lead to differential abundance of tRF isoforms, reported in many disease studies we reviewed here.

In an example of yet another functional mechanism, a tRF was found to affect apoptosis and unwind mRNA of ribosomal proteins RPS28 and RPS15, thereby enabling their translation and regulating ribosome biogenesis [21]. While most of the studies reviewed here considered tRFs interacting with their targets in the context of the RISC, some of them reported other modes of action, such as direct binding to proteins [22].

Since their discovery, tRFs have been implicated in range of cellular functions and diseases, including cell proliferation [23], global regulation of RNA silencing [24,25], inflammation [26], cell signaling [27], and immunity [28]. They show great promise as potential biomarkers for multiple human conditions, paving the way for exciting new avenues in disease research and therapeutic development [29]. However, the level of scrutiny and biological evidence supporting tRF function greatly varies between different studies. Often, such results leave gaps in our understanding of the function of these emerging regulators and some unsubstantiated claims ultimately might lead other researchers down the wrong paths, as specialists in disease research may have less experience with interpreting small RNA data. This has motivated us to review the recent literature on tRFs in the disease context and also to classify the reviewed papers by the level of evidence provided in support of their claims. Here, we concentrated on the current literature describing various roles of tRFs in different diseases, particularly focusing on four large areas: cancer, neurological, cardiovascular, and musculoskeletal disorders.

## 2. Naming tRFs and Classifying Paper/Study Types

Since neither of the naming schemes above was predominantly favored in the reviewed studies, we decided to use a simpler naming method in this review. We refer to a tRF by a very short ID, such as **T[REF]** or **T[REF]a** (**T** followed by a **[REF]**erence number of the paper describing the tRF and a **letter** indicating that there are several tRFs in the reference). We list these IDs in Table A1 in Appendix A, which also provides the tRF sequences we found in the reviewed papers. This makes a navigation between the studies and sequences very easy within our review. One can use the **[REF]** part in the ID to navigate to the reference or search for “**T[REF]**” to obtain to the sequence.

### Evolution of tRF Detection and Analysis

The early methods used to detect tRFs primarily relied on RT-PCR and Northern blot analysis. These traditional approaches demanded prior knowledge to formulate and test various hypotheses related to tRFs, which were then largely considered to be random breakage products and poorly understood. As research progressed, there were significant improvements in sequencing technology, particularly with the development of next-generation sequencing (NGS) methodologies. These innovations have greatly enhanced the reliability and efficiency of detecting a wider range of tRFs that were previously overlooked or unrecognized. Many of these fragments were initially dismissed as mere background noise in the data and regarded as nonfunctional.

However, the significance of these fragments became increasingly evident following the observations of their loading to AGO and potential function via seed region binding [4,6,8,16,17], akin to miRNAs, which play a crucial role in cellular regulation. With the abundance of sequence data now available through NGS, it has become entirely feasible for researchers to explore these fragments in depth. This has led to a better understanding of their biological relevance and implications in various physiological and pathological processes. The evolving landscape of tRF research illustrates the transformative effect of technological advancements in genomics, enabling scientists to unveil previously hidden layers of genetic regulation and function.

Yet the ease of obtaining NGS data in many cases results in oversimplified interpretation of such data and excessive reliance on predictive tools, computational annotation, and lack of validation, as illustrated in this review. Following this path, the vast majority of studies reviewed here assume the RISC-based mode of action of tRFs, an unnecessary limitation.

**Figure 2:**
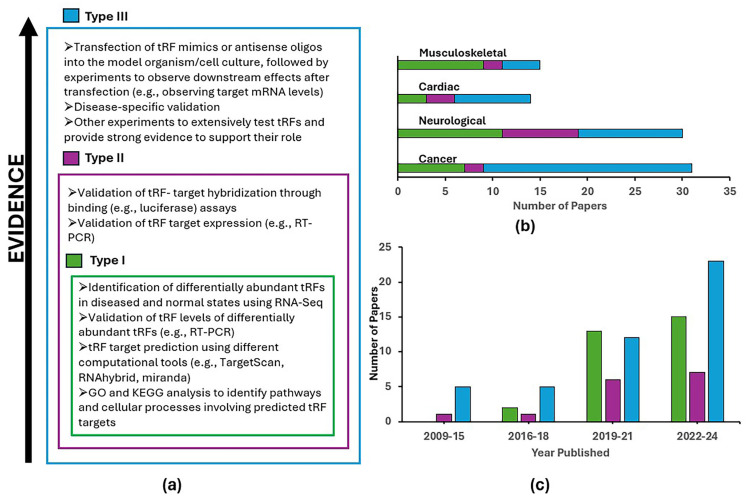
Study types observed. (**a**) The colored boxes display the proposed classification of paper types. Each box is outlined in a color that corresponds to a paper type; (**b**) the stacked bar plot indicates the number of papers by type discussed in this review for the four disease groups (shown above their corresponding bar); (**c**) the number of sampled disease area primary (not review) paper types published over the past 15 years. Colors in the plots match the paper types shown in (**a**).

We selected ~100 papers using search terms from the respective disease areas and the words “tRF”, “tRNA derived” or “tRNA fragment(s)”. We reviewed papers discussing the roles of tRFs in different diseases and categorized them into three types based on the level of experimental validation conducted to establish these roles (Figure 2).

Most studies begin with RNA-sequencing (RNAseq) data to identify differentially abundant tRFs between diseased and normal states, followed by validation of these tRF levels using techniques like qRT-PCR. Although tested tRFs are often claimed to be randomly chosen, randomization approaches are typically never described, and most chosen targets get confirmed. So, how do these three types of studies follow up on such initial RNAseq and qRT-PCR findings of different abundance of tRFs?

Type I papers typically use prediction tools, such as TargetScan [30], RNAhybrid [31], and miRanda [32], to identify putative targets of identified tRFs and then follow up with Gene Ontology (GO) or Kyoto Encyclopedia of Genes and Genomes (KEGG) analyses to further speculate on pathways and cell processes involving predicted targets. However, these predictive tools have been developed for miRNA targets and even for miRNA they have high false prediction rates. Therefore, such results should be taken with great caution, and additional validation experiments are necessary to confirm the predicted targets and functions of tRFs. Although these papers might indicate the role of tRFs in diseases, they have significant limitations, as the predictions only hypothesize the role of the tRFs and do not provide further confirmation that the interactions with named targets really take place and under what conditions. This group of papers mostly ignores the variety of tRF mechanisms mentioned above in Section Examples of Mechanisms and Processes Involving tRFs.

Type II papers take their analyses a step further by validating specific interactions of tRFs using wet lab techniques. For example, they may employ luciferase assays to confirm regulatory interactions between tRFs and their gene targets (likely via tRF-target hybridization). Additionally, some type II studies validate the expression of the targets at mRNA level (e.g., using qRT-PCR) or as proteins (e.g., Western-blots). This is important for supporting hypotheses of the RISC-based action of tRFs on their predicted targets but does not exclude other mechanisms.

Type III papers further build on such results by extensively testing tRFs to verify their roles beyond the computational binding predictions and binding assays. For instance, these studies often include additional disease-specific validation, in vitro and in vivo testing, often involving transfection of tRF mimics or antisense oligos, and other experiments that provide further compelling evidence to elucidate the roles of tRFs. Ultimately, these papers strongly establish the actual significance of particular tRFs in specific disease contexts and explore their potential therapeutic applications, emphasizing the role of tRFs in future medical developments. While there is a somewhat alarming trend of the increasing type I papers in the past decade, the dominant growth of type III studies is encouraging (Figure 2c), as they provide increasing evidence on tRF functions in health and disease.

## 3. Cancer

### 3.1. tRFs in Pancreatic Cancer

In pancreatic cancer, exosome-derived T[33]a has been found to promote liver metastasis by upregulating WDR1 gene (WD repeat-containing protein 1), which interacts with actin, activating hepatic stellate cells and mediating the infiltration of myeloid-derived suppressor cells to form pre-metastatic niches [33]. Similarly, T[34]a and T[35]a have been demonstrated to enhance the cell proliferation, migration, and invasion of pancreatic cancer cells, suggesting that tRFs play a key role in the aggressive nature of this disease [34,35]. Specifically, T[35]a promotes malignancy in pancreatic adenocarcinoma by inhibiting transcription factor *ASCL2*, regulating downstream genes [35]. Further, specific tRFs that could be developed into promising diagnostic markers for pancreatic cancer have been identified; T[36]a and T[36]b had particularly high diagnostic accuracy for the disease [36].

### 3.2. tRFs in Lung Cancer

In lung cancer, one study revealed that certain tRFs are significantly dysregulated and associated with tumor progression; in particular, T[37]a was touted as a biomarker for predicting prognosis from an early stage of cancer [37]. Other studies also highlighted the dysregulation of tRFs in lung cancer, especially T[38]a, T[38]b, T[38]c, and T[38]d [38,39]. A more recent paper identified three tRFs that were downregulated in lung cancer tissues (T[40]a, T[40]b, and T[40]c), providing evidence that tRFs may play multiple roles in lung cancer proliferation [40].

### 3.3. tRFs in Colorectal Cancer

In colorectal cancer (CRC), tRFs play possible roles in regulating tumor suppression and stem-like characteristics. According to Huang et al. [41], T[41]a plays a crucial role in suppressing cancer stem cell-like properties and metastasis in CRC by downregulating the Notch-signaling pathway through *JAG2* (jagged canonical Notch ligand 2) targeting. Another study demonstrated that tRFs purified from non-pathogenic *Escherichia coli* strains exhibit cytotoxic effects on CRC cells. These effects are enhanced with 2′-O-methylation modifications, suggesting potential for microbiome-derived therapeutic applications [42]. Chen et al. [43] also identified that some tRFs are significantly upregulated in CRC tissues, correlating with advanced disease stages and metastasis risk, highlighting their role as possible prognostic biomarkers. Additionally, tRFs have been linked to metabolic pathways like vitamin metabolism and cyclic GMP signaling, which are critical to CRC progression and suggest diverse regulatory roles for these molecules [44]. It was further speculated that tRFs provide high diagnostic accuracy, perhaps surpassing traditional biomarkers like CEA and CA199, thus suggesting their utility in early CRC detection [45].

### 3.4. tRFs in Gastric Cancer

In gastric cancer (GC), specific tRFs have demonstrated tumor-suppressive properties by modulating key signaling pathways and gene expression. For instance, T[46]a acts as a tumor suppressor in GC by inhibiting the PTEN/PI3K/AKT pathway, thereby reducing cell proliferation and migration [46]. Another tRF highlighted is T[47]a, which exerts anti-cancer effects in GC by downregulating G-protein coupled receptor *GPR78*, limiting tumor growth and metastasis in vivo [47]. Similarly, T[27]a was found to suppress GC progression by binding to AGO2 and inhibiting STAT3 signaling, which is critical for tumor proliferation and survival [27]. Additionally, it has been noted that T[47]a impedes GC cell invasion and migration through the WNT and MAPK pathways [48]. In contrast to the tumor-suppressive roles of the aforementioned tRFs, T[49]a has been identified as a promoter of GC progression. This tRF enhances the proliferation, migration, and invasion of GC cells by targeting acyl-coenzyme A dehydrogenase short/branched chain (*ACADSB*), a gene implicated in lipid metabolism and ferroptosis regulation. The suppression of *ACADSB* by T[49]a contributes to lipid accumulation and reduced ferroptosis, thereby facilitating tumor growth and metastasis [49].

### 3.5. tRFs in Nervous System Cancers

Research on tRFs in nervous system cancers, especially brain tumors, remains limited despite their potential as biomarkers for diagnosis and prognosis. T[50]a, T[50]b, and T[50]c were significantly reduced in diffuse gliomas, and their decreased abundance was associated with poor survival. They may exert their influence by modulating the expression homeobox gene *HOXA13* and *RBM43* (RNA Binding Motif Protein 43), known to contribute to glioma progression [50].

The role of T[51]a and T[51]b has been further examined to reveal prospective biomarkers and treatment targets for glioma. Functional enrichment study revealed that the target genes of these two tRFs are involved in the regulation of blood vessel formation, inhibiting glioma cell proliferation, migration, and in vitro vasculogenic mimicry creation. T[51]b could act as a tumor suppressor in glioma cells by targeting the 3′UTR of *S100A11* (calcium-binding protein that plays a role in cell growth, inflammation, and cancer) mRNA, thereby regulating glioma development [51]. A study exploring ANG and its ability to cleave tRFs demonstrated that tRFs inhibit protein synthesis and promote the assembly of stress granules independently of eIF2α phosphorylation [52].

Given the results in gliomas, it is expected that comparable insights can be gained from examining tRF roles in glioblastoma, the most aggressive malignant primary brain tumor. Differential abundances between glioblastomas and low-grade gliomas linked upregulated tRFs like T[53]a, T[27]a, T[53]b, T[53]c, and T[53]d to pathways like axon guidance and DNA repair [53], which are critical in cancer development.

### 3.6. tRFs in Breast Cancer

The studies on tRFs include their mechanistic roles in breast cancer and possible diagnosis and prognosis biomarkers. A study found that T[54]a was significantly elevated in the plasma of breast cancer patients and discussed relative merits of using a panel of three tRFs (T[54]a, T[54]b, T[54]c) versus any of the individual tRFs as biomarkers [54]. Another study identified that T[55]a binds to the RNA-binding protein NCL and may promote *p53* mRNA translation, possibly inhibiting cell proliferation [55]. T[56]a, T[56]b, T[56]c, and T[56]d were shown to interact with the RNA-binding protein, YBX1, indicating their possible role in post-transcriptional regulation via destabilization of YBX1-bound transcripts, crucial for tumor growth and metastasis. By competitively displacing these transcripts from YBX1, the tRFs effectively suppress the expression of key oncogenes. The downregulation of oncogenes stabilized by YBX1, such as *EIF4G1* and *AKT1*, is linked to reduced proliferation and invasive capabilities of breast cancer cells [56]. Furthermore, T[41]a, downregulated by 25-hydroxyvitamin D, 25(OH)D, promotes breast cancer progression by interfering with MBNL1 (muscleblind-like splicing regulator 1)-mediated RNA splicing and JNK pathway regulation. Its upregulation enhances tumor proliferation and stemness, while 25(OH)D-mediated suppression mitigates these effects, highlighting its potential as a therapeutic target in breast cancer [57].

### 3.7. tRFs in Ovarian Cancer

Similarly to breast cancer, some studies have revealed tRFs as modulators of key molecular pathways during ovarian cancer progression. T[58]a, originating from the Chinese yew, suppresses ovarian cancer growth via downregulation of *TRPA1*, a calcium channel sensitive to oxidative stress [58]. In high-grade serous ovarian cancer, T[59]a drives increased cell growth, spread, and invasion by downregulating the tumor-suppressor *HMBOX1* and stimulating pathways, such as MAPK and WNT [59].

### 3.8. tRFs in Prostate Cancer

The regulation of tRNA methylation appeared to be a significant factor in controlling the tumor cell growth in prostate cancer (PCa). One key enzyme involved in this mechanism is tRNA methyltransferase METTL1, which modulates the methylation of tRNA and affects cellular processes in tumor cells [60]. METTL1 has been shown to affect PCa cells downstream of the AKT-signaling pathway [60,61]. To further support the role of tRNA in cancer, sex-hormone-dependent tRNA-derived RNAs (SHOT-RNAs) have been identified in PCa, where the sex-hormone-signaling pathways activate ANG cleavage of amino acylated mature tRNAs, resulting in the accumulation of SHOT-RNAs, causing cell proliferation, and promoting tumorigenesis [10].

To further corroborate the function of tRNA in the regulation of PCa, ELAC2 has been established as a risk factor for increasing the susceptibility to the disease [2]. ELAC2 plays a role in the biogenesis of short RNAs in the tRF-1 series [62]. The tRFs predominantly localize in the cytoplasm [24], consistent with the cytoplasmic localization of ELAC2 [63,64].

### 3.9. Leukemia

A comparative study has shown the roles of tRFs and miRNAs in the regulation of gene expression in chronic lymphocytic leukemia (CLL) [38]. The study highlighted key interactions, including miR-15/16 targeting the oncogene *BCL2* [65], and tRFs, such as T[38]d, targeting the oncogene *TCL1* [38], which is critical in CLL pathogenesis. In another study, T[23]a was found downregulated in lymphoma cell lines and primary biopsies, with possible significance in regulating endogenous target genes such as *RPA1* [23]. This is an essential gene (the largest subunit of the Replication Protein A complex) for many aspects of DNA dynamics, such as genome replication and its deregulation may lead to changes in the proliferation rate [66].

These studies showcase the significance of tRFs in cancer biology, highlighting various tRFs that are differentially abundant between cancerous and normal states. They also suggest the involvement of tRFs in regulating key pathways, tumor proliferation, apoptosis, and metastasis. Type III papers, in particular, contributed to findings that establish tRFs’ role in cancer and provide insights into potential therapeutic targets for treatment. As research advances, profiling tRFs in cancer holds promise for identifying novel biomarkers, aiding treatment, and facilitating early detection, emphasizing the need for continued exploration in this field.

## 4. Neurological Diseases

### 4.1. Spinal Cord Injury and tRFs

Trauma is the primary cause of spinal cord injury (SCI). The development of promising treatments for patients with SCI necessitates a focus on the fundamental molecular mechanisms of the SCI-induced secondary injury. In the aftermath of a SCI, the body undergoes a response involving the release of tRFs, that appear essential for cell repair and recovery from inflammation [67]. Several signaling pathways exhibit interrelations with intracerebral hemorrhage, including the modulation of the G protein-coupled receptor signaling pathway, endocytosis, and response to oxidative stress as a result of tRF-mRNA interaction [68]. To preliminarily ascertain the potential functions of putative tRFs, studies utilizing contusion SCI model research indicated that four tRFs may suppress *BDNF* (brain-derived neurotrophic factor) and modulate the pathophysiological process through the MAPK and neurotrophin-signaling pathways. It was determined through the luciferase reporter that T[69]a may affect the regulation of the post-injury pathophysiologic processes [69]. In the later phases of post-injury, tRFs may contribute to neural regeneration by influencing pathways that promote axonal growth and synaptic plasticity. This can facilitate the restoration of sensory and motor functions [67].

### 4.2. MELAS and tRFs

Mitochondrial encephalomyopathy, lactic acidosis, and stroke-like episodes (MELAS) is an oxidative phosphorylation (OXPHOS) disease that primarily affects the nervous system and muscles. It is a mitochondrial genetic disorder that is inherited maternally, but it can also be caused by sporadic mutations in mitochondria (mt). The cybrid model of MELAS was employed to find that the m.3243A > G substitution in mtDNA leads to OXPHOS dysfunction. The m.3243A > G site belongs to tRNA LeuUUA and its tRF, containing the mutated site was significantly upregulated in MELAS compared to WT cells, while the same tRF with an unmutated site was significantly downregulated. Change in predicted targets between unmutated and mutated tRFs was noted but not validated [70]. A later study of the same group showed that both the oxygen consumption rate and levels of transcripts encoding for OXPHOS Complex I subunits significantly increased in MELAS cells transfected with the mimic of the unmutated tRF but did not change for the mutated mimic [71].

### 4.3. Autism and tRFs

Autism spectrum disorder is a neurodevelopmental disorder caused by a combination of genetics and environmental factors, including infection-associated maternal immune activation (MIA) during pregnancy [72]. The placenta of C57BL/6 mouse strains was employed to investigate the function of tRFs in the maternal–fetal interface during gestation. This investigation concentrated on the variations in tRF levels that occur during the fetus’s development in response to MIA. Significant upregulation of tRFs, such as T[73]a, T[73]b, T[73]c, and T[73]d, occurred after 3 h of maternal immune activation in fetal development [73].

### 4.4. Traumatic Brain Injury and tRFs

Traumatic brain injury (TBI) represents a significant public health issue worldwide, characterized by external trauma that results in both functional disruptions and pathological changes within the brain [74]. Post-TBI, upregulated tRFs suppress synaptic function genes (*LRRC4C*, *KCTD3*, and *MORF4L2*), resulting in impaired vesicle-mediated transport and trans-synaptic signaling. Conversely, downregulated tRFs result in upregulation of vesicle-associated membrane protein VAMP8 and C-C ligand CCL4, respectively, both of which contribute to increased inflammation [75]. Another study showed upregulation of T[76]a and T[76]b, correlated with worse behavioral outcomes in chronic neuroinflammation. Notably, T[76]a targets *CPLX1*, a gene essential for vesicle exocytosis and neurotransmitter release regulation. Altered *CPLX1* expression may contribute to neuronal loss and TBI pathophysiology, suggesting that these tRFs also influence chronic inflammation and hinder recovery after TBI [76]. The analysis of tRF abundance profiles in experimental TBI mice revealed that treatment with Xuefu Zhuyu Decoction (XFZYD) significantly restored these profiles to a state similar to that of healthy controls. This may link the action of XFZYD with tRFs and their potential targets. T[77]a, T[77]b, T[77]c, and T[77]d were downregulated in the TBI group but upregulated with the XFZYD treatment. Expression of their predicted target genes, such as Liprin-alpha *PPFIA3*, RAS family member RAB11A, Ring Finger Proteins RNF216, and RNF6, showed an inverse pattern to these tRFs. Of note, *RNF6*, targeted by T[77]d, contributes to inflammation and neuronal recovery in TBI [77].

### 4.5. Stroke and tRFs

Stroke is a widespread medical condition affecting millions globally each year and remaining the second leading cause of death, according to the World Health Organization. Recent research suggests that altered tRFs may regulate key signaling pathways involved in neuronal recovery post-stroke, highlighting their potential role in addressing this ongoing global health challenge [78,79]. A study reported significant changes in small RNAs (sRNAs) after ischemic stroke, with a decrease in miRNA levels and an increase in tRFs targeting cholinergic transcripts (genes involved in the production or regulation of acetylcholine, a key neurotransmitter in the nervous system). Six tRFs (T[79]a, T[79]b, T[79]c, T[35]a, T[34]a, and T[79]d) were upregulated in stroke patients. Analysis of transcription factors in monocytes revealed two clusters targeted either by miRNAs or tRFs. T[79]b specifically targeted the transcript of Z-DNA-binding protein 1 [79]. tRFs were explored as novel therapeutic targets of Buyang Huanwu Decoction (BYHWD), a traditional Chinese medicine with a possible role in neuroprotection and promoting neurogenesis used to treat neurological impairments and disabilities caused by stroke. In a study, the BYHWD group showed improved outcomes as compared to the control groups, where three tRFs (T[80]a, T[80]b, and T[80]c) were upregulated after BYHWD treatment [80]. tRFs might also be useful in other stroke contexts as potential biomarkers for distinguishing acute Ischemic Stroke, Intracerebral Hemorrhage, and Subarachnoid Hemorrhage, and advancements in detection technologies could enable rapid point-of-care tests for quicker diagnoses, reduced delays, and improved outcomes [81].

### 4.6. Perioperative Neurocognitive Disorder and tRFs

Perioperative neurocognitive disorder (PND) is a significant postoperative complication, particularly in elderly patients, characterized by cognitive impairments, such as memory deficits, anxiety, and reduced exploratory behavior. In aged mouse models, dysregulated tRF abundance was observed in hippocampal neurons, with tRFs particularly enriched in pathways associated with neuronal development and ferroptosis regulation. Among these, T[82]a was shown to be capable of suppressing ferroptosis in primary neurons. It promoted the expression of protective ferroptosis-related proteins such as GPX4 and FTH1, while reducing oxidative damage markers like Fe^2+^ and malondialdehyde upon transfection of the tRF mimic. While the mechanism remains unknown, the modulation of ferroptosis by this tRF highlights its therapeutic potential as a treatment to reduce cognitive problems in PND [82].

### 4.7. Alzheimer’s Disease and tRFs

tRFs appear to play important roles in Alzheimer’s disease (AD). A study focused on T[83]a and T[83]b revealed their significantly elevated levels in the hippocampal tissues of AD patients. This increase in tRF abundance may affect the brain’s oxidative stress responses, which further damage neurons. Additionally, lower levels of NSUN2, an enzyme that stabilizes tRNAs and makes tRNAs more susceptible to cleavage by ANG, resulting in the overproduction of tRFs [83]. T[84]a has been shown to accumulate in the mitochondria of glutamatergic neurons, impairing mitochondrial translation and disrupting cristae organization. This process reduces glutaminase-dependent glutamate biosynthesis, increasing cognitive decline. Moreover, this mitochondrial dysfunction reduces synaptosomal glutamate levels, leading to defects in synaptic organization and memory decline. Disruptions caused by tRFs have been shown to contribute to age-related defects in mitochondrial cristae organization and glutamate metabolism. Experimental evidence suggests that targeting this tRF with antisense oligonucleotides, may help in reversing these phenotypes [84]. Furthermore, a paper found that female AD patients showed lower levels of cholinergic targeting tRFs weakening cholinergic function, thereby contributing to memory loss and learning difficulties [85]. In addition, Klotho knockout models demonstrated elevated levels of T[86]a, which was found to directly interact with spliceosome-associated proteins and components of the RNA degradation machinery. These interactions suggest a role for this tRF in modulating RNA splicing and mRNA decay, processes that are crucially impaired in neurodegenerative diseases like AD. Moreover, a deficiency of aging-related Klotho resulted in transcriptomic changes that mirrored alterations observed in AD, including dysregulation of genes involved in amyloid-beta processing and synaptic plasticity [86].

### 4.8. Huntington’s Disease and tRFs

In Huntington’s disease (HD), specific tRFs are emerging as both contributors to neurodegeneration and potential early biomarkers for the disease. T[87]a has been identified as highly neurotoxic within the HD-affected part of the brain. Experimental studies revealed that this tRF induces neuronal death, inflammation, and motor deficits when introduced in models, closely replicating the characteristic neuropathology of HD. When introduced into primary neuronal cultures, this tRF significantly reduced cell viability, indicating its neurotoxic effects. Additionally, in vivo experiments involving the injection of this tRF into the striatum of wild-type mice resulted in selective neuronal death in the striatum, increased markers of neuroinflammation, and disruptions in motor function. Behavioral tests confirmed motor deficits in the treated mice, closely mirroring the progressive motor impairments observed in HD patients [87]. Specific plasma extracellular vesicle tRFs, such as T[88]a and T[88]b, are found to be downregulated in HD mutation carriers at premanifest stages, holding promise as biomarkers, potentially aiding in the early detection and monitoring of disease progression [88].

### 4.9. Parkinsons and tRFs

In Parkinson’s disease (PD), specific tRFs have been shown to exhibit distinct abundance patterns linked to disease pathology. A study utilizing the senescence-accelerated mouse prone 8 model of age-related neurodegenerative diseases identified dysregulated tRFs in the brain that potentially contribute to PD progression, T[89]a and T[89]b [89]. Another study reported that tRFs levels differ between male and female patients. RNA-seq was used to identify specific tRFs associated with PD and showed that these tRFs display distinct abundance patterns in PD patients compared to healthy controls [90].

### 4.10. Epilepsy and tRFs

Epilepsy is a neurological condition marked by frequent, unprovoked seizures, frequently brought on by irregular electrical activity in the brain. Seizures often result in severe cellular stress, which may elevate tRF levels, such as T[91]a, T[91]b, and T[91]c. These tRFs were more prevalent in pre-seizure samples but not in post-seizure samples, indicating that they might be used as biomarkers of seizure risk in individuals with epilepsy [91]. The comparison of pre-seizure and post-seizure samples obtained from electrochemical methods from patients showed T[91]b and T[91]c may be potential biomarkers for seizure [92].

### 4.11. Amyotrophic Lateral Sclerosis and tRFs

Amyotrophic Lateral Sclerosis (ALS) is characterized by the accumulation of misfolded proteins leading to motor neuron death. T[93]a secreted by the neuronal cells associated with the increased angiogenin expression and slower disease progression in ALS, suggesting a prognostic value in ALS patients with slow disease progression [93]. A recent study considered the role of T[94]a in ALS pathophysiology using transcriptomics and proteomics, suggesting its inhibitory effects on protein synthesis machinery, aligning with stress granule-independent regulatory mechanisms. T[94]a may influence energy metabolism and synaptic function, pathways critical to ALS progression [94]. Similarly, a study indicated that specific tRFs cleaved by angiogenin in ALS patients interact with Y-box binding protein 1 (YB-1). YB-1 is a translational repressor that hampers translation initiation and promotes stress granule assembly formation. Additionally, the study highlights that DNA analogs of the tRFs form a G4 structure, enabling them to enter motor neurons and activate a YB-1-dependent neuroprotective response, which in turn inhibits stress granule assembly triggered by the tRFs [95].

Research on tRFs in neurological diseases has revealed their role in essential neuronal processes such as neuronal signaling and synaptic plasticity. Type III papers connect tRFs with potentially regulated mRNAs associated with neurodegeneration, inflammation, and stress responses. They also demonstrate that changes in tRF levels could contribute to the pathology of neurological diseases. Ongoing research into the specific roles and mechanisms of tRFs in neurological contexts will aid in uncovering novel biomarkers and therapeutics.

## 5. Cardiovascular Diseases

### 5.1. Cardiac Hypertrophy, Heart Failure and tRFs

Cardiac hypertrophy, a condition that can lead to heart failure, is a major contributor to global morbidity and mortality and is commonly associated with cardiovascular conditions such as cardiomyopathies [96]. The levels of T[97]a and T[97]b in the hypertrophic myocardium differed significantly compared to the control group in the isoproterenol (ISO)-induced cardiac hypertrophy model. Cardiomyocytes’ surface area increased following the forced increase in T[97]a and T[97]b using synthetic mimics, and the expression of the related hypertrophy markers *ANF*, *BNP*, and *β-MHC* was elevated. Additionally, the luciferase reporter assay verified that T[97]a may target the 3′UTR of *TIMP3*, a hypertrophic regulator, and inhibit its expression [97]. Further, the role of tRFs in pathological cardiac hypertrophy (PCH) was explored by sequencing tRFs in plasma samples from PCH patients and healthy controls, finding 4185 tRFs with significant differences in abundance, although the sheer number makes one question their common role. T[98]a was considered a possible diagnostic biomarker, as injecting its mimic in hypertrophic cells effectively reduced cell size and decreased levels of hypertrophy markers, including natriuretic peptides/cardiac hormones ANP and BNP [98].

### 5.2. Pulmonary Arterial Hypertension and tRFs

Pulmonary arterial hypertension (PAH) is a potentially fatal condition exhibiting characteristics similar to those of cancer [99]. The pathological foundation of PAH involves irregular apoptosis, fibrosis, cellular proliferation, and metabolic dysregulation within pulmonary arterial smooth muscle cells (PASMCs) [99,100]. A small RNA microarray analysis revealed 816 significantly dysregulated tRFs in the plasma of PAH patients compared to healthy controls. Six were validated, with T[101]a and T[101]b upregulated, and T[101]c, T[101]d, and T[101]e downregulated. The authors suggested that the upregulation of T[101]b in the peripheral venous blood of PAH patients may affect abnormal proliferation and anti-apoptosis of PASMCs by targeting the BMPR2-signaling pathway and used as a biomarker [101].

A role of 8-oxoguanine modifications was proposed in pulmonary hypertension. T[102]a has been shown to play a role in PASMC proliferation and resistance to apoptosis. This modification enhances PH pathogenesis by targeting and inhibiting the expression of key genes, such as *WNT5A* and *CASP3*, which are pivotal in regulating vascular remodeling and cellular homeostasis [102].

### 5.3. Atherosclerosis and tRFs

Atherosclerosis (AS) is considered as the most common type of cardiovascular disease and has a significant impact on global health. AS condition involves buildup of fatty substances and the formation of plaque in the artery, restricting blood flow and leading to chronic inflammation [103,104]. Findings from recent studies have revealed involvement of tRFs in AS. Some tRFs are upregulated in AS and difference in the abundance was used to suggest that T[103]a may serve as a biomarker or a target for treatment [103]. The link between AS and tRFs was further explored in a recent study by using salvianolic acid B (SalB), which is known in the treatment of cardiovascular conditions. T[88]a and T[105]a were identified as possibly involved in the MAPK pathway, and their levels were lower in the AS group but significantly upregulated after being treated with SalB. Changes were also observed in the inflammatory cytokine levels. Compatible with these findings, the target genes *SRF* (serum response factor), arrestin beta *ARRB*, and interleukin 1 receptor associated kinase *IRAK4* showed upregulation after treatment suggesting that tRFs play a role in processes that leads to atherosclerosis [105].

### 5.4. Rheumatic Heart Disease and tRFs

Rheumatic heart disease (RHD) is a significant global health concern, especially in developing countries where RHD is often associated with atrial fibrillation (AF), a condition that leads to irregular and fast beating of the heart’s upper chambers [106]. Recent studies have shed light on tRFs’ potential roles in the pathogenesis of RHD. A study highlighted the differential abundance of tRFs in RHD patients with and without AF, identifying specific tRFs, such as T[107]a, that may play a role in regulating gene expression related to AF [107]. Another tRF, T[108]a, is upregulated in AF and may contribute to its progression by promoting ferroptosis and by targeting and downregulating cystine/glutamate transporter protein SLC7A11, which mediates the progression of AF [108].

### 5.5. Aortic Dissection and tRFs

tRFs have emerged as regulatory molecules in cardiovascular diseases like aortic dissection. The T[109]a and T[110]a have shown significant differential levels, and studies support the role of these tRFs in controlling the proliferation and migration of vascular smooth muscle cells (VSMCs), which are central to the disease progression. T[109]a was found to be upregulated in aortic dissection and linked to enhanced proliferation, migration, and phenotype switching of VSMCs. T[110]a was shown to be downregulated in both human and animal models. In addition, it targets the signaling via transcription factor STAT4, a pathway involved in VSMC differentiation and survival. As a consequence, tRFs might mitigate their pro-inflammatory and pro-proliferative effects, suggesting a protective role [109,110]. Furthermore, their detection in bodily fluids, such as plasma, underscores their utility in non-invasive diagnostics and offers the potential for biomarker development.

### 5.6. Myocardial Ischemia and tRFs

In myocardial ischemia, tRFs are emerging as key regulators of cellular responses to ischemic injury. Study of the cardioprotective effects of caloric restriction in ischemic models identified T[111]a and T[111]b that may play a role in reducing ischemic damage by modulating inflammatory and metabolic pathways [111]. These findings align with the work by Li et al., which showed that tRFs like T[112]a and T[83]a inhibit angiogenesis, a crucial process for post-ischemic recovery [112]. The tRFs in atherosclerosis may interfere with pathways of tRFs in ischemic heart disease [103,111].

### 5.7. Intimal Hyperplasia and tRFs

The role of tRFs in intimal hyperplasia and vascular remodeling in cardiovascular diseases is highlighted by two key fragments: T[113]a and T[114]a. T[113]a promotes VSMC proliferation and migration, which are crucial in the progression of intimal hyperplasia. It achieves this by downregulating the FAS cell surface death receptor, enhancing VSMC survival and growth following arterial injury [113]. Similarly, T[114]a contributes to pathological angiogenesis, a process also implicated in cardiovascular diseases, by suppressing vasohibin-1 (*VASH1*), leading to increased endothelial cell proliferation and migration [114]. These fragments link to vascular complications, such as atherosclerotic plaque neovascularization and ischemic conditions. Targeting these tRFs may offer innovative therapeutic approaches to control intimal hyperplasia, vascular remodeling, and angiogenesis-related complications in cardiovascular diseases.

The roles of tRFs in cardiac disease underscore their involvement in heart development and function. Evidence from type III papers illustrates their regulatory capacity in processes such as cardiac hypertrophy, cell proliferation, migration, and fibrosis. The findings above also indicate that tRFs can modulate important pathways responsible for inflammation and metabolism. Further investigation is needed to understand the role of tRFs in cardiac disease, as they hold significant potential to treat these conditions, highlighting their importance in overall cardiovascular health.

## 6. Musculoskeletal Diseases

tRFs have recently been found in the context of osteoarthritis, osteoporosis, fibrous dysplasia, and sarcopenia [115,116,117,118]. They are often proposed as potential biomarkers since, in some musculoskeletal diseases, their higher (T[116]a) or lower abundance (T[117]a) is detected, depending on the specific indication [116,117]. An investigation of the expression profiles of tRFs in the serum of enthesitis-related arthritis (ERA) patients found significantly increased levels of T[119]a in ERA compared to healthy patients, naming it a promising candidate biomarker [119]. tRFs are also involved in the regulation of cellular metabolism and inflammation, known to be critical in the pathophysiology of osteoarthritis [116]. Inflammatory stimuli were observed to alter the abundance of T[120]a, suggesting it might have a useful role in the management of RA [120]. Other authors highlighted the differences in circulating tRF levels between patients with rheumatoid arthritis (RA) and psoriatic arthritis (PsA). RA patients had lower, while PsA patients had higher, levels of the same set of tRFs. Further in the study, in IL-1 receptor antagonist knockout mice, tRF levels were seen altered in arthritic conditions. T[119]a was proposed as a potential biomarker for enthesitis-related arthritis that may help enhance early detection and improve patient outcomes by differentiating this condition from other inflammatory disorders [119].

In osteoarthritis, T[121]a was claimed to be relevant in regulating autophagy and cartilage degeneration by targeting the mTOR signaling pathway. In support of this claim, expression data were used to demonstrate differences in levels of this tRF in disease and healthy cartilage, complemented with evidence of its impact on mTOR pathway proteins and autophagy markers, such as microtubule-associated protein 1 light chain 3 gene *LCB3* and autophagy-related *BECLIN1* by Western-blot and immunofluorescence [121].

Research on T[122]a has demonstrated that it influences the metabolism of anterior cruciate ligament cells by modulating the expression of the inhibitor of nuclear factor kappa-B kinase IKBKB, which underscores the significance of tRFs in ligament health and recovery from injuries [122]. tRFs have been shown to post-transcriptionally regulate gene expression in chondrocytes exposed to inflammatory stimuli such as *IL-1ß*, highlighting their possible role in mediating inflammatory responses in cartilage [120]. Emerging studies suggest that tRFs play roles in supporting tissue repair in muscles and reducing oxidative stress in bone, impacting musculoskeletal health.

At the intersection with cancer, another study emphasized tRFs as potential biomarkers in multiple myeloma, a hematological malignancy that affects the bone marrow. It was observed that loss of T[123]a was linked to a higher risk of short-term disease progression following chemotherapy. Also, this tRF was suggested to influence NOTCH and ERBB3 pathways, involved in cell proliferation and survival [123]. Another example is T[124]a, which seem to regulate the tumor suppressor *NELL2* [124]. tRFs can interact with RNA-binding proteins, isolating the latter from their target RNAs. For instance, T[125]a was suggested to influence the stability of mRNAs by sequestering insulin-like growth factor mRNA-binding protein IGF2BP1, which is crucial for maintaining the stability of certain transcripts. Another example is a well-known transcription factor, MYC, which is critical in regulating cell proliferation, differentiation, and apoptosis [125]. Reports of tRFs effects on other musculoskeletal diseases are relatively recent. One study identified differentially expressed tRFs that correlate with growth defects from overgrowth syndrome or from intrauterine growth restriction [126,127]. T[126]a inhibits the expression of insulin-like growth factor *IGF1*, leading to developmental effects on porcine skeletal muscle cells, such as muscle atrophy and reduced cell proliferation [126]. In a study of osteosarcoma, a malignant cancer with high invasion and metastasis, six tRFs were identified as potential diagnostic biomarkers, and early detection was a key factor for survival. However, there were limitations due to the low incidence of osteosarcoma, resulting in shortage of samples [128].

A methyltransferase METTL1 (mentioned above in the context of prostate cancer) catalyzes m7G modification of tRNA, which is cleaved to generate a mitochondrial tRF, involved in chondrocyte degeneration. This tRF has been demonstrated to bind the 3′UTR of mRNA *SENP1*, a regulatory enzyme for SUMO1 modification and to influence the PINK1/PARKIN pathway leading to mitochondrial dysfunction and chondrocyte apoptosis. Lowering SENP1 expression leads to SIRT3 SUMOylation, reducing sirtuin SIRT3 activity and inhibiting PINK1/PARKIN and any mitophagy-related proteins. The inhibition of PINK1/PARKIN-mediated mitophagy that begins by a cascade caused by the tRF will eventually lead to decreased mitochondrial membrane potential, increased ROS, and a metabolic shift towards glycolysis. This pathway highlights how T[129]a can disrupt energy metabolism and expedite chondrocyte degeneration [129].

In addition to musculoskeletal disorders, there is interest in studying tRFs in osteogenesis as their levels change in certain conditions, causes. tRFs in circulation in a closed tibial fracture showed elevated T[130]a during fracture healing [130]. tRFs have been shown to promote muscle regeneration, early inflammatory response during time of injury, and myoblast differentiation [131].

Musculoskeletal disease research on tRFs seems currently dominated by type I studies, and we hope this situation will be improving. Type II and III papers further highlight the regulatory role of tRFs in various musculoskeletal disorders by identifying potential targets and validating them, respectively. As more type III papers emerge, they enhance understanding of the association between tRFs and the disease, suggesting diagnostic and therapeutic potential for early detection and improved recovery for patients with these disorders.

## 7. Conclusions

As demonstrated above, tRFs represent a significant emerging superclass of small RNA molecules that go beyond our traditional understanding of tRNA function. The diversity of tRF classes, their unique biogenesis pathways, and the properties of these classes underscore the unknown breadth of their potential functions in various cellular processes. Their ability to modulate gene expression and influence cellular behaviors highlights their importance in cell biology and medical applications. They play multifaceted roles across many diseases, demonstrating their potential as biomarkers and therapeutic targets/agents. In cancers, tRFs are involved in critical processes such as tumor proliferation and suppression, metastasis, and regulation of key signaling pathways. In cases of neuronal or muscular injury and cardiovascular diseases, tRFs can target multiple genes affecting various recovery and inflammatory pathways, which can either promote or inhibit healing or disease progression.

The advantage of considering multiple papers is that identical tRFs from unrelated studies may be noticed. We observed several cases when the same tRF was implicated in two or more different disease contexts. Most of these studies were of type II and III, thus likely deserving further investigation. We are currently finalizing a manuscript describing their overlapping roles and potential implications.

Aided by advancements in sequencing technologies, research of tRFs is entering a phase of certain maturity. However, inconsistencies in nomenclature and classifications still pose challenges for researchers, making cross-study comparisons difficult. Tools like tDRnamer and MINTbase “license plates” or the tatDB naming approach provide means of standardizing identification of these molecules, yet each method has its own limitations. Future endeavors in this field should focus on establishing (and adhering to) a unified nomenclature system and fostering collaborative efforts to compile comprehensive databases that capture the full spectrum of tRFs together with their targets.

In this review, we introduced a system of classifying papers based on the extent of validation performed to verify the roles of specific tRFs and their targets in the context of different diseases. This provides a framework for unifying the standards in tRF studies to extract the real value from these combined efforts and pass it on to disease researchers.

Given the promising correlational data between tRF abundance and disease stage or patient outcomes, further stringent experiments are essential for elucidating their underlying mechanisms and enhancing their applicability in clinical settings. Certain tRFs have shown promise as diagnostic markers, offering potential high accuracy for early detection and prognosis. Their diverse regulatory roles underscore the need for further studies to fully harness their potential as tools for the diagnosis and treatment of a wide set of diseases. Overall, tRFs represent a novel but rapidly maturing area of exploration that could lead to significant advancements in multiple disease areas and personalized medicine, if stringent standards are maintained.

## Data Availability

Not applicable.

## Appendix A

**Table A1:** tRF sequences, their corresponding ID and reference. Sequences were extracted from the paper text whenever possible and otherwise by a good-faith effort from Figures and Supplementary Materials (in some papers, nothing was found). Sequences are colored by the corresponding paper type (type 1-green, type 2-purple, and type 3-blue). Boldface indicates disease areas.

Sequence	ID	Reference
**Cancer**		
TCCCTGGTGGTCTAGTGGTTAGGTT	T[33]a	[33]
ATCCCACCGCTGCCACCA	T[34]a	[34]
TCCCCGGCACCTCCACCA	T[35]a	[35]
CTTACACTTAGGAGATTTCAACTTAACTTGACCGCTCTGAC	T[36]a	[36]
CCCCGAAAATGTTGGTTATACCCTTCCCGTACTACCA	T[36]b	[36]
GGCCGGTTAGCTCAG	T[37]a	[37]
TTGTGGAAACAATGGTACGGCAAGGGCCTCTTT	T[38]a	[38]
AAGGGAGGTTATGATTAACTTT	T[38]b	[38]
GCTAAGGAAGTCCTGTGCTCAGTTT	T[38]c	[38,39]
CCGTGTTTCCCCCACGCTTT	T[38]d	[38,39]
GAAGCGGGTGCTCTTATTTTTT	T[40]a	[40]
TCGAGAGGGGCTGTGCTCGCAAGGTTTCTTTT	T[40]b	[40]
AAGTGGTTCCCGTTTT	T[40]c	[40]
TCCCACCGCTGCCACCC	T[41]a	[41]
GTTTCCGTAGTGTAGTGG	T[46]a	[46]
TAGGATGGGGTGTGATAGGTGGCA	T[47]a	[47,48]
GCATGGGTGGTTCAGTGGTAGAATTCTCGCCTG	T[27]a	[27]
GCTTCTGTAGTGTAGTGGTTATC	T[49]a	[49]
CAACTATCTTATTCTCCTTT	T[50]a	[50]
ATCCCCTTCTGACACCA	T[50]b	[50]
TCGAATCCCACTTCTGACACCA	T[50]c	[50]
GCATTGGTGGTTCAGTGGTAGAATTCTCGCCTC	T[53]a	[53]
GCATGGGTGGTTCAGTGGTAGAATTCTCGCCTG	T[27]a	[53]
GCCGTGATCGTATAGTGGTTAGTACTCTGCG	T[53]b	[53]
CACTGTAAAGCTAACTTAGCATTAACCTTTTA	T[53]c	[53]
GCATTGGTGGTTCAGTGGTAGAATTCTCGCCTG	T[53]d	[53]
GGCCGCGTGGCCTAATGGA	T[51]a	[51]
GGGCCAGTGGCGCAATGGA	T[51]b	[51]
AGAGGGTCTTTTTCACCCCGCTGTTGCTCTTT	T[54]a	[54]
TCGAGAGGGGCTGTGCTCGCAAGGTTTCTT	T[54]b	[54]
GCCGAAATAGCTCAGTTGGGAGAGCGTTAGACTG	T[54]c	[54]
AGGCGGCCCGGGTTCGACTCCCGGTGTGGGAA	T[55]a	[55]
AGTGGTTAGGATTCGGCGCTCTCACCGCCGCGGCCCGCGTTCGTTTCGCGGTCAGCCCA	T[56]a	[56]
AGTGGTTAGTATCCCCGCCTGTCACGCGGGAGACCGGCGTTCAATTCGGCGACGACCA	T[56]b	[56]
AGTGGTTAGCATAGCTGCCTTCCAAGCAGTTGACACGGGTTCGATTGGCGACCAACGCACCA	T[56]c	[56]
GTGAATCCTTCGATAGCTCAGTTGGTAGAGCGGAGGACTGTAGTTGGCTGTGTGCTTAGACATCCTTAGGTCGCTGGTTCGAATCCG	T[56]d	[56]
TCCCACCGCTGCCACCC	T[41]a	[57]
ACCTGGTGAACCGATGCAGGCG	T[58]a	[58]
Sequence not directly provided ^1^	T[59]a	[59]
TCGATTCCCGGCCAATGCACCA	T[23]a	[23]
**Neurological**		
GTCCGCTCTTAAGATGGTGACTTGGTGGGTACG	T[69]a	[69]
TTCCGGCTCGAAGGACCA	T[73]a	[73]
TCTCGCTGGGGCCTCC	T[73]b	[73]
ATCCTGCCGACTACGCCA	T[73]c	[73]
ATCACGTCGGGGTCACCA	T[73]d	[73]
TCGCGGGTTCGATCCCCGTACGGGCCAC	T[76]a	[76]
TCCAGGGTTCAAGTCCCTGTTCGGGCGC	T[76]b	[76]
TCGACTCCCGGTATGGGAACCA	T[77]a	[77]
CAAATCTCGGTGGAACCTCCA	T[77]b	[77]
TCGAGCCCCAGTGGAACCACCA	T[77]c	[77]
TCGAATCCCACCGCTGCCACCA	T[77]d	[77]
TCGATTCCCGGCCAATGCACC	T[79]a	[79]
TCGATCCCCGGCATCTCCACCA	T[79]b	[79]
TCCCCGGCACCTCCACCA	T[35]a	[79]
ATCCCACCGCTGCCACCA	T[34]a	[79]
TCCCCGGCATCTCCACCA	T[79]c	[79]
TCAATCCCCGGCACCTCCACCA	T[79]d	[79]
GGGGGATTAGCTCAAA	T[80]a	[80]
ATGGACTGCTAATCCATTGTGCTCT	T[80]b	[80]
TCCCACATGGTCTAGCGGTTAGGATTCCT	T[80]c	[80]
GCCCGGCTAGCTCAGTCGGTAGAGCATGGGAC	T[82]a	[82]
GCATTGGTGGTTCAGTGGTAGAATTCTCGCCT	T[83]a	[83]
TCCCTGGTGGTCTAGTGGTTAGGATTCGGCGCT	T[83]b	[83]
TCCCTGGTGGTCTAGTGGTTAGGATTCGGCGCTC	T[84]a	[84]
TAACCGAAAGGTTGGTGGTTCGAGCCCAACCCAGGGACGC	T[86]a	[86]
GGGGGTGTAGCTCAGTGGTAGAGCGCGTGC	T[87]a	[87]
TCCCTGGTGGTCTAGTGGTTAGGATTCGGCG	T[88]a	[88]
GCATTGGTGGTTCAGTGGTAGAATTCTCGC	T[88]b	[88]
GTGGTGTGCTAGTTAATTT	T[89]a	[89]
CCTGTCACGCGGGAGACCGGGGC	T[89]b	[89]
GCATGGGTGGTTCAGTGGTAGAAT	T[91]a	[91]
TCCCTGGTGGTCTAGTGGTTAGGATT	T[91]b	[91,92]
GGGGATGTAGCTCAGTGGTAGAGC	T[91]c	[91,92]
GTTTCCGTAGTGTAGTGGTTATCACGTTCGCCTC	T[93]a	[93]
GCATTGGTGGTTCAGTGGTAGAATTCTCGCCTGC	T[94]a	[94]
**Cardiovascular**		
GCAATGGTGGTTCAGTGGTAGAATTCTCGC	T[97]a	[97]
TCCCATATGGTCTAGCGGTTAGGATTCCTGGTTT	T[97]b	[97]
CTGAATCCAGCGATCCAGTT	T[98]a	[98]
TTCCCTGACGGGGAGCCA	T[101]a	[101]
CCTCACACGCGAAAGGTCCCCGGT	T[101]b	[101]
TAGTGTAGTGGTCATCACGTTCGCC	T[101]c	[101]
CCGTGATCGTATAGTGGTTAGTACTCTGC	T[101]d	[101]
GTCGGTAGAGCATGGGA	T[101]e	[101]
GCCGGGTACTTTCGTATTTT	T[102]a	[102]
Sequence not directly provided	T[103]a	[103]
TCCCTGGTGGTCTAGTGGTTAGGATTCGGCG	T[88]a	[105]
GCATTGGTGGTTCAGTGGTAGAATTCTC	T[105]a	[105]
GCCCGGCTAGCTCAGTCGGTAGAGCATGGGACTCT	T[107]a	[107]
GCGTTGGTGGTATAGTGGTGAGC	T[108]a	[108]
AGTAAGGTCAGCTAAATAAGCTATCGGGCC	T[109]a	[109]
GCAGTCAAATGCTCTACCACTGAGCTATACCCCC	T[110]a	[110]
TGAATCTAACAACAGGAAATCAAATTCCTTATTTACCCA	T[111]a	[111]
GCGTTGGTGGTATAGTGGTGAGCATAGCT	T[111]b	[111]
GTTTCCGTAGTGTAGTGGTTATCACGTTCGCCT	T[112]a	[112]
GCATTGGTGGTTCAGTGGTAGAATTCTCGCCT	T[83]a	[112]
ACTCTGGACTCTGAATCT	T[113]a	[113]
TCCCTGGTGGTCTAGTGGTTAGGATTTGGCG	T[114]a	[114]
**Musculoskeletal**		
TCCCTGGTGGTCTAGTGGCTAGGATTCGGCGCTT	T[116]a	[116]
GTAGTCGTGGCCGAGTGGTTAAGGC	T[117]a	[117]
CGATTCCCGGCCAATGCACCA	T[119]a	[119]
ACCTCCCCCGTGGGCCT	T[120]a	[120]
Sequence not directly provided	T[121]a	[121]
GGCTCCATAGCTCAGGGGT	T[122]a	[122]
TGAATCTGACAACAGAGGCTTACGACCCCTTA	T[123]a	[123]
AGCCCCAGTGGAACCACCA	T[124]a	[124]
GGTTCCATGGTGTAATGGTTAGCACTCTGGACT	T[125]a	[125]
TCCCATATGGTCTAGCGGTTAGGATTCCTGGT	T[126]a	[126]
TCAATTCCTCTTCTTAACACCA	T[129]a	[129]
Sequence not directly provided	T[130]a	[130]

^1^ In certain papers, we could not find a specific tRF sequence. We avoided making inferences regarding indirectly referred sequences like PCR primers, etc.
